# Glofitamab Treatment in Relapsed or Refractory DLBCL after CAR T-Cell Therapy

**DOI:** 10.3390/cancers14102516

**Published:** 2022-05-20

**Authors:** Vera Rentsch, Katja Seipel, Yara Banz, Gertrud Wiedemann, Naomi Porret, Ulrike Bacher, Thomas Pabst

**Affiliations:** 1 Department of Medical Oncology, Inselspital, Bern University Hospital, 3010 Bern, Switzerland; vera.rentsch@students.unibe.ch; 2 Department of Biomedical Research, University of Bern, 3008 Bern, Switzerland; katja.seipel@dbmr.unibe.ch; 3 Institute of Pathology, Inselspital, University of Bern, 3008 Bern, Switzerland; yara.banz@pathology.unibe.ch; 4 Center of Laboratory Medicine (ZLM), Inselspital, Bern University Hospital, 3010 Bern, Switzerland; gertrud.wiedemann@insel.ch (G.W.); naomiazur.porret@insel.ch (N.P.); 5 Department of Hematology, Inselspital, Bern University Hospital, 3010 Bern, Switzerland; veraulrike.bacher@insel.ch

**Keywords:** CAR T-cell therapy, glofitamab, diffuse large B-cell lymphoma (DLBCL), relapse

## Abstract

**Simple Summary:**

CAR T-cell therapies represent a major advance in the treatment of relapsed B-cell non-Hodgkin lymphomas. Nevertheless, a significant proportion of these patients will experience disease progression following CAR T treatment. For these patients, no standard therapeutic procedure is established so far. The novel bispecific antibody glofitamab has shown promising activity in the treatment of refractory or relapsed B-cell non-Hodgkin lymphomas. In this study, we provide evidence for good tolerance and promising efficacy of glofitamab administration in patients relapsing after CAR T-cell therapy.

**Abstract:**

Chimeric antigen receptor T-cells (CAR T) treatment has become a standard option for patients with diffuse large B-cell lymphomas (DLBCL), which are refractory or relapse after two prior lines of therapy. However, little evidence exists for treatment recommendations in patients who relapse after CAR T-cell treatment and the outcome for such patients is poor. In this study, we evaluated the safety and efficacy of a monotherapy with the bispecific CD20xCD3 antibody glofitamab in patients who progressed after CAR T treatment. We report nine consecutive patients with progressive DLBCL after preceding CAR T-cell therapy. The patients received a maximum of 12 cycles of glofitamab after a single obinutuzumab pre-treatment at an academic institution. CRS was observed in two patients (grade 2 in both patients). We observed an overall response rate of 67%, with four patients achieving a complete response and a partial remission in two patients. Interestingly, we identified increased persistence of circulating CAR T-cells in peripheral blood in three of the five patients with measurable CAR T-cells. Our data suggest that glofitamab treatment is well tolerated and effective in patients with DLBCL relapsing after CAR T-cell therapy and can enhance residual CAR T-cell activity.

## 1. Introduction

The most common type of aggressive non-Hodgkin Lymphomas is diffuse large B-cell lymphoma (DLBCL) [1]. Whereas most patients achieve a complete remission following first-line therapy with chemotherapy and rituximab, approximately 40% will, ultimately, relapse [2,3]. Such patients usually undergo salvage therapy, with a proportion of 30–40% of them responding [4,5], and these patients are candidates for consolidation with autologous stem cell transplantation (ASCT). Among them, up to 50% may relapse after ASCT [6,7,8]. For DLBCL patients who relapse or are refractory after at least two lines of prior therapy, treatment with chimeric antigen receptor T-cells (CAR T) has become the standard option [9,10]. Registration studies reported an overall response rate (ORR) of 52–82% after CAR T treatment, with complete response (CR) rates of 40–52% [11,12,13]. Remarkably, real-world studies reported comparable outcomes [14,15,16]. Clinical response rates were reported to correlate with expansion levels and the duration of persistence of CAR T-cells [17,18]. In addition, varying outcomes after CAR T therapy seem to be associated to different DLBCL subtypes [19].

However, along with the increased use of this innovative therapy, the number of patients relapsing after CAR T-cell treatment is emerging as a novel challenge. Various approaches are being explored for patients with relapses following established CD19 targeted CAR T-cell therapy, e.g., CD19-specific CAR T-cells that express a PD-1/CD28 chimeric switch receptor [20]. So far, the survival of patients relapsing after CAR T therapy is poor [21,22], and treatment recommendations for these patients are largely lacking [23,24]. Treatment with bispecific antibodies is a prominent option, and recent studies reported ORR ranging from 60% to 90%, with CR rates between 40% and 60% [25]. Importantly, bispecific antibody treatment also appears to be effective in patients relapsing after CAR T-cell therapy [26,27,28]. Among these compounds, glofitamab is a bispecific T-cell engager (BiTEs) consisting of two variable fragments joined by a flexible glycine-serine linker. They express two binding domains, with one of them recognizing CD3 on T-cells and the other binding to the target tumor antigen CD20 on (malignant and normal) B-cells. Thereby, immune response is enhanced and effector T-cells are directed to exert cytotoxicity against cells bearing the target antigen [29]. Glofitamab differs from other bispecific antibodies by its 2:1 configuration, which mediates enhanced anti-lymphoma efficacy and a prolonged half-life [30].

In the first in-human phase I dose-escalation study NP30179 (NCT03075696), the recommended phase II dose (RP2D) of increasingly applied 2.5 mg/10 mg/30 mg dose steps was established. The study reported an ORR of 65.7% and a CR rate of 57.1%, while toxicities were manageable. The most common adverse event was cytokine release syndrome (CRS), occurring in 50.3% of the patients [31]. CRS is the result of a hyperactivated immune response and is associated with enhanced production of inflammatory cytokines, including interleukin (IL)-6 [32]. The IL-6-receptor antagonist tocilizumab and steroids are used as standard treatment against CRS [33].

Apart from the promising results in phase I studies with glofitamab, its availability off the shelf makes it an attractive option as a rescue treatment after CAR T failure. In addition, due to its targeted mode of action, it has the potential to avoid toxic effects on residual circulating CAR T-cells’ possible enabling synergistic effects. In this retrospective study, we analyzed the safety and efficacy of administering monotherapy with glofitamab in DLBCL patients relapsing after CAR T treatment, and we specifically investigated the effects of glofitamab treatment on the remaining CAR T-cells in the peripheral blood.

## 2. Materials and Methods

In this single-center study, we retrospectively analyzed real-world experiences of consecutive DLBCL patients receiving glofitamab monotherapy after CAR T treatment at the Inselspital, University Hospital in Bern, Switzerland. Glofitamab was provided free as part of the standardized pre-approval access program (PAA) of the manufacturing company (Roche Pharma), as the compound is not yet approved in Switzerland. We included the data of all consecutive patients with relapsed or refractory DLBCL after CAR T treatment, who received at least one dose of glofitamab monotherapy as the next line after CAR T treatment at our institution between September 2020 and March 2022. All patients gave written informed consent, and the study was approved by a decision of the local ethics committee of Bern, Switzerland.

The treatment comprised pretreatment with a single dose of obinutuzumab (Gazyvaro^®^) 1000 mg in cycle 1 on day 1 (C1D1), thereby depleting B-cells in the peripheral blood and lymphoid organs resulting in reduced T-cell activation and cytokine release [30]. In order to mitigate eventual side effects, glofitamab was applied using a standard step-up dosing mode. The first dose of 2.5 mg was applied on day 8 of cycle 1 (C1D8) and the second dose (10 mg) was given on day 15 of cycle 1 (C1D15). The first cycle lasted 21 days. From day 1 of cycle 2 (C2D1) until day 1 of cycle 12 (C12D1) 30 mg of glofitamab was applied every 21 days. The initial doses of glofitamab on C1D8 and C1D15 were administered to adequately pre-hydrated patients as 4 h infusions. In the absence of infusion-related symptoms, subsequent doses of glofitamab were given as 2 h infusions.

The Ann Arbor classification was used to define initial tumor stages while risk assessments were determined by the International Prognostic Index (IPI). Tissue biopsies served to assess tumor antigens such as CD20 positivity using routine immunohistochemistry (IHC) and to verify lymphoma histology. Bone marrow infiltration was assessed by standard aspiration and biopsy. Lymphomas that occupied at least one-third of the chest width or that had a diameter larger than 7 cm were assessed as bulky diseases.

We measured the occurrence of CAR T-cell-specific DNA in the peripheral blood by digital-droplet PCR (ddPCR) technology [31,33]. Peak IL-6 and C-reactive protein (CRP) serum levels were recorded during the treatment, in order to evaluate the occurrence of CRS and infections [32]. Toxicities were graded using CTCAE 4.0 criteria (Common Terminology Criteria for Adverse Events). Remission status was based on CT or PET assessments and classified by the RECIST 1.1 criteria.

All statistical analyses were performed using GraphPad Prism^®^ Version 8. Progression-free survival (PFS) was defined as the time between C1D1 and death, progression, or last follow-up, whichever occurred first. Overall survival (OS) was defined as the time between C1D1 and death from any cause or last follow-up. Survival curves were estimated using the Kaplan–Meier method and differences were evaluated by the log-rank test. Patients being alive and progression free were censored at the time of the last follow-up.

## 3. Results

### 3.1. Clinical Characteristics of the Patients

Between September 2020 and March 2022, nine patients with CAR T failure were treated with a monotherapy of glofitamab at our institution. All of them had DLBCL, relapsing after previous CAR T treatment. DLBCL was transformed in five cases (here, the limited patient number has to be seen) from follicular lymphoma (FL) and in one patient from marginal zone lymphoma (MZL). At first diagnosis, four patients had stage IV disease, three patients had stage III, and two patients had stage II disease. All lymphomas were high-intermediate risk or high risk. Detailed patient characteristics at diagnosis are summarized in Table 1.

Table 2 reports patient characteristics at the start of glofitamab treatment. The median age at glofitamab therapy was 66 years, and the median time from diagnosis to the beginning of the treatment was 3.8 years. Patients had a median of three previous lines of therapy, which included a median of three prior anti-CD20 therapies. All patients underwent previous CAR T-cell therapy as the last preceding line of treatment. The median time between CAR T-cell infusion and glofitamab treatment was six months. The median peak expansion of CAR T-cells was 3260 copies/μg DNA after CAR T treatment. In contrast, the median level of circulating CAR T-cells before the first glofitamab administration was reduced to 34 copies/μg DNA. In three (33%) patients, no circulating CAR T-cells were detectable at all. After CAR T-cell therapy, complete response (CR) was achieved in two patients, six patients were in partial response (PR) and one patient had stable disease (SD). Before glofitamab therapy, all patients had PET-CT-documented relapsed DLBCL, and relapsing disease was verified in six (67%) of them.

### 3.2. Therapeutic Course and Safety

All nine patients completed at least one cycle of glofitamab treatment. Two patients had ongoing progression and ultimately died after the first cycle due to lymphoma progression. Lymphoma progression led to cessation of glofitamab treatment in one patient each after cycles 3, 9, and 10. Four (44%) patients completed the planned 12 cycles. Treatment characteristics are listed in Table 3.

Glofitamab was generally well tolerated, and there were no discontinuations or dose reductions due to treatment-related adverse events (AEs). All AEs that occurred during the treatment period are summarized in Table 3. The majority of the patients (7 of 9) experienced fatigue, three of them regularly during the first few days after the infusion of the medication.

**Figure 1:**
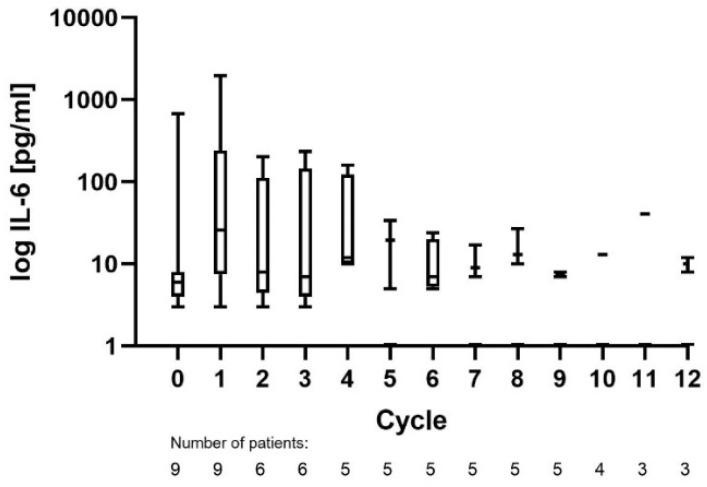
Peak IL-6 measurements of all treated patients during each cycle.

CRS was observed in two patients during cycle 1, both being grade II. One patient was treated with tocilizumab and dexamethasone. Biomarkers for CRS, such as IL-6 and CRP, were elevated in cycle 1, with median peak levels of 43 pg/mL and 131 mg/L. Peak IL-6 values during all cycles are shown in Figure 1.

One patient experienced symptoms of tumor lysis syndrome after the first administration of obinutuzumab, with emesis, diarrhea, and abdominal pain for 2 days. Transient neutropenia occurred in three patients (grade 1 in one patient and grade 3 in two patients). One patient each experienced anemia (grade 1) and thrombocytopenia (grade 1), conditions that were not previously known. Other AEs included obstipation (grade 1) in four patients, diarrhea in three patients (grade 1 in two patients and grade 2 in one patient), nausea (grade 2) in four patients, emesis (grade 1) in one patient, dyspnea in two patients (grade 1 in one patient and grade 2 in one patient), and cough (grade 1) in two patients.

Infections with at least one febrile episode of ≥38 °C were detected in six patients, while the median number of days with fever was only one day. Infections, with at least one identified germ, were seen in four patients, three of which were viral (Enterovirus (two patients), Varicella Zoster Virus (one patient)). One infection was bacterial (Staphylococcus), detected by blood culture.

### 3.3. Efficacy of Glofitamab Therapy

All patients were evaluable for response, and response rates are summarized in Table 4. The overall response rate after glofitamab treatment was 67%. CR assessed by PET-CT was achieved in four patients and partial response (PR) was seen in two patients. We had stable disease (SD) in one patient and progressive disease (PD) in two patients. The median time between the first treatment infusion and CR was 8.3 months. One patient achieved CR after only cycle 1 of the treatment. In three patients with ultimate CR, PR occurred first. Achievement of PR was documented after cycles 2 or 3, after a median of 56 days. Two patients with PR relapsed after a median of 142 days after the first Glofitamab infusion and, therefore, terminated the treatment prematurely.

**Figure 2:**
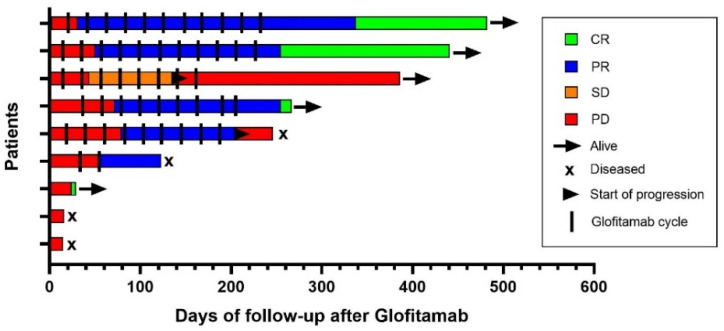
Swimmer plot illustrating outcomes after glofitamab therapy.

During the study period, four deaths occurred. All patients died due to lymphoma progression, and two of them received only one cycle of treatment. The median time from treatment beginning until death was 70 days. A summary of treatment outcomes is depicted in Figure 2.

**Figure 3:**
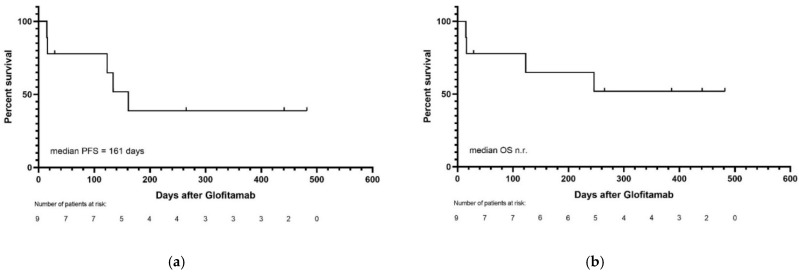
(**a**) Progression-free and (**b**) overall survival of all patients.

The median time until the last follow-up was 246 days. Response at the last follow-up was CR in four patients and PD in five patients. Progression-free survival (PFS) was 44%, and 5/9 (56%) of the patients were alive at the last follow-up. The median PFS was 161 days, and median OS was not reached. PFS and OS are presented in Figure 3.

### 3.4. Effects of Glofitamab on CAR T-Cells

All nine patients had previously received CAR T-cell therapy. The kinetics of CAR T-cell-specific DNA in peripheral blood was assessed before, during, and after glofitamab treatment. One patient died before the measurement of CAR T-cells in the peripheral blood during cycle 1. In the three patients without detectable CAR T-cells before the therapy, CAR T-cells in the peripheral blood remained undetectable during glofitamab therapy. The decrease in CAR T-cells after CAR T-cell therapy continued following glofitamab in two patients. However, three patients experienced a re-expansion of CAR T-cells in the peripheral blood after glofitamab infusions. Peak CAR T-cell expansion by digital-droplet PCR (ddPCR) occurred after a median of 35 days from the start of glofitamab treatment. Later, during glofitamab treatment, the initially enhanced cell expansion decreased again; however, it did not fall below the levels seen before the start of glofitamab therapy.

**Figure 4:**
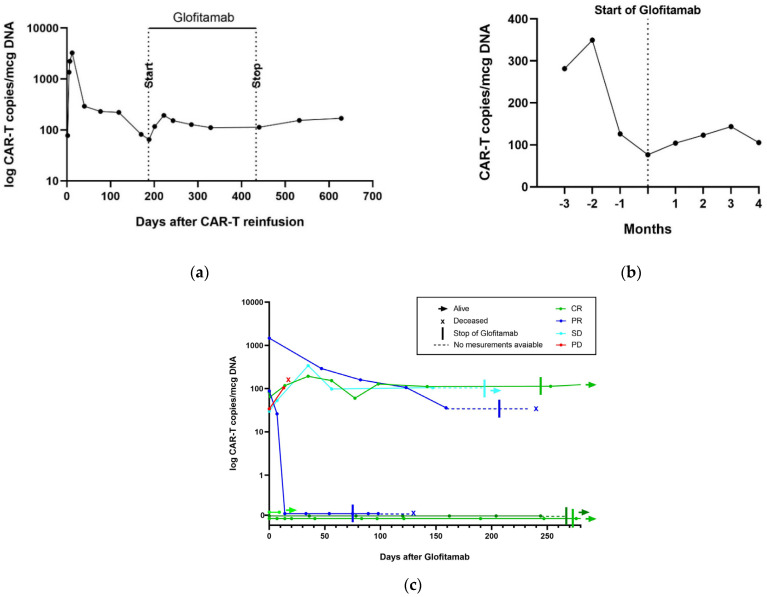
CAR T-cell expansion during glofitamab therapy. (**a**) CAR T-cell copies/mcg DNA in one patient who had CR and experienced a re-expansion of CAR T-cells after glofitamab; (**b**) median CAR T-cell copies/mcg DNA before and after glofitamab in all patients who had measurable CAR T-cells; (**c**) CAR T-cell copies/mcg DNA for each patient after the administration of glofitamab.

The median peak CAR T-cell expansion of all evaluable patients during glofitamab treatment was 66 copies/μg DNA. No association between CAR T-cell expansion and response to glofitamab could be observed, as three patients with CR did not have any detectable CAR T-cells before and after treatment. Only one patient with CR experienced a CAR T-cell expansion during glofitamab therapy. CAR T-cell expansion after glofitamab is illustrated in Figure 4. Given the small numbers, any correlation between CAR T-cell levels and response during glofitamab treatment remains speculative at this moment.

## 4. Discussion

In this study, we investigated patients with refractory or relapsed DLBCL, who received glofitamab as the next line of treatment, after relapse following CAR T treatment, at a single academic institution. All patients were heavily pre-treated and had exhausted the available options. Our study intended to provide information on the safety of glofitamab after CAR T-cell infusion, preliminary evidence of efficacy, and the ability of glofitamab to eventually enhance the declining activity of residual CAR T-cells.

In our study cohort, glofitamab application turned out to be safe and was generally well tolerated. We did not record unexpected new adverse events during glofitamab therapy. In particular, the incidence of infections and the observed germs, as well as the rate of glofitamab-related neutropenia and thrombocytopenia, were comparable to the phase I dose-escalation study. In contrast, the occurrence of CRS was significantly lower in our cohort with 22%, as compared to 71% in the phase 1 study [31]. CRS after glofitamab treatment does not seem to be associated with persisting CAR T-cells, as one patient out of two patients with CRS in our cohort did not have persisting CAR T-cells in the peripheral blood at all. Furthermore, no CRS was observed in patients with enhanced circulating CAR T-cell levels.

Our data suggest that glofitamab therapy resulted in significant clinical response in a subset of patients with DLBCL relapsing after CAR T treatment. This supports earlier reports of the efficacy of BsAbs [25]. Furthermore, our results propose that BsAbs can be effective in patients who experienced CAR T failure, supporting previous reports on mosunetuzumab and odronextamab, which are other CD3/CD20 BiTE antibodies [27,28,34]. Schuster et al. reported a CR rate of 22.2% in 18 patients treated with mosunetuzumab who relapsed after CAR T-cell therapy and had at least 3 months of follow-up [27], while Bannerji et al. reported a CR rate of 27% in 30 patients treated with odronextamab after CAR-T failure [28].

To our knowledge, no information on the efficacy of glofitamab administration in DLBCL patients relapsing after CAR T-cell therapy is yet available. The ORR and CR rates in our cohort after glofitamab treatment were similar to those observed in the phase I dose-escalation trial of glofitamab, in which patients achieved an ORR of 65.7% with a CR rate of 57.1% when treated with the RP2D [31]; however, most of these patients had no CAR-T therapy.

The median OS in DLBCL patients progressing after CAR-T treatment is poor. Two studies reported overall survival of 161 and 180 days only [20,21]. Our data suggest that glofitamab therapy can prolong survival in patients failing CAR T therapy, since we observed a median OS not reached after a median follow-up of 246 days. However, our data need longer follow-up and confirmation in larger series of patients, preferably in a prospective comparative study. Even though the results of our retrospective analysis seem promising, it will be necessary to investigate the administration of Glofitamab after failed CAR T-cell therapy in a larger number of patients in prospective studies. A French phase II study (NCT04703686) is investigating glofitamab in patients with relapsed or refractory non-Hodgkin lymphoma after CAR T therapy [35]. This study and other investigations are needed to establish recommendations for patients with progressive DLBCL after CAR T-cell therapy.

Finally, we found evidence that Glofitamab administration may enhance circulating CAR T-cells in the peripheral blood assessed by ddPCR. This effect was similarly observed following mosunetuzumab treatment [27], but has not been described so far for glofitamab. Among the patients with re-expansion of CAR T-cells in our study, only one achieved CR, making it impossible to draw conclusions between CAR T-cell re-expansion and response. However, given the relevance of the level of CAR T-cell expansion and persistence for the effectiveness of CAR T-cell therapy [17,18], it is tempting to speculate that the effectiveness of glofitamab treatment after CAR T failure may, in part, be due to its potential to induce CAR T-cell re-expansion. One may hypothesize that this could be due to lymphoma burden reduction and thereby, antigen reduction, leading to the proliferation of “less exhausted” CAR T-cells. However, we favor the concept that the main therapeutic effect of glofitamab in this patient series is due to CD3 cytotoxicity, rather than residual CAR T cells, as the efficacy of glofitamab was not limited to patients with CAR-T cell expansion in the peripheral blood following glofitamab application.

## 5. Conclusions

In conclusion, treatment with glofitamab in patients with refractory or relapsed DLBCL after CAR T-cell therapy seems to be safe and effective. Additionally, our data suggest that the administration of glofitamab can lead to an expansion of residual CAR T-cells. However, larger studies are needed to assess whether this effect contributes to the responses observed and in order to propose an improved management of patients after CAR T-cell failure.

## Figures and Tables

**Table 1:** Patient characteristics at diagnosis.

Patient Characteristics		
Age, years, median (range)	60	(41–73)
Gender, male, *n* (%)	5	56%
Histology Diffuse large B cell lymphoma (DLBCL), *n* (%)	9	100%
De Novo, *n* (%)	3	33%
Transformed DLBCL, *n* (%)	6	64%
-from follicular lymphoma (FL), *n* (%)	5	56%
-from marginal zone lymphoma (MZL), *n* (%)	1	11%
Elevated LDH, *n* (%)	9	100%
Disease Stage, *n* (%)		
II	2	22%
III	3	33%
IV	4	44%
IPI, *n* (%)		
3 (high-intermediate risk)	5	56%
4–5 (high risk)	4	44%
Bone marrow infiltration, no of pts (%)	5	56%
Infiltration, % of cells, median (range)	50	(10–90)
Bulky disease, *n* (%)	2	22%
CNS infiltration, *n* (%)	0	0%
B symptoms, *n* (%)	1	11%
Immunohistochemistry CD20+, *n* (%)		100%

Elevated LDH: Lactate dehydrogenase > 480 U/L; IPI: International prognostic index; CNS: Central nervous system.

**Table 2:** Patient characteristics at start of Glofitamab therapy.

Patient Characteristics		
Age at glofitamab therapy, median (range)	66	(41–75)
Time from diagnosis to glofitamab, months, median (range)	45	(9–284)
Previous therapy lines before glofitamab, median (range)	3	(2–7)
Anti CD-20 therapies before glofitamab, median (range)	3	(1–6)
Previous autologous stem cell transplantation, *n* (%)	5	(56%)
Previous CAR T cell therapy, *n* (%)	9	100%
tisagenlecleucel, *n* (%)	6	67%
axicabtagene ciloleucel, *n* (%)	3	33%
CAR T toxicities, *n* (%)		
CRS	6	67%
Neurotoxicity	2	22%
Best response to CAR T cell therapy, *n* (%)		
SD	1	11%
PR	6	67%
CR	2	22%
Interval CAR T-cell infusion to glofitamab, days, median (range)	187	(86–655)
Peak expansion of CAR T cells, copies/μg DNA, median (range)	3260	(54–12,578)
CAR T cell expansion before glofitamab, copies/μg DNA, median (range)	34	(0–1470)
Patients without detectable CAR T cells before glofitamab, *n* (%)	3	33%
Bulky disease, *n* (%)	2	22%
CNS infiltration, *n* (%)	1	11%
B symptoms, *n* (%)	3	33%

CAR T: Chimeric antigen receptor T-cells; CRS: Cytokine release syndrome; SD: Stable disease; PR: Partial response; CR: Complete response; CNS: Central nervous system.

**Table 3:** Glofitamab treatment characteristics and adverse events.

Treatment Characteristics		
Glofitamab given, *n* (%)	9	100%
Number of cycles completed, *n* (%)		
1 cycle	2	22%
3 cycles	1	11%
9 cycles	1	11%
10 cycles	1	11%
12 cycles	4	44%
Premature termination of treatment, *n* (%)	5	56%
-due to lymphoma progression, *n* (%)	5	
Peak IL-6 after glofitamab, pg/mL, median (range)	43	(7–1975)
Peak expansion of CAR T cells after glofitamab, copies/μg DNA, median (range)	66	(0–340)
**Adverse events, *n* (%)**		
CRS	2	22%
-Grade II	2	
Neurotoxicity	0	0%
Tumor lysis syndrome	1	11%
Anemia	1	11%
Neutropenia	3	33%
Thrombocytopenia	1	11%
Fatigue	7	78%
Nausea	4	44%
Emesis	1	11%
Diarrhea	3	33%
Obstipation	4	44%
Dyspnea	2	22%
Cough	2	22%
At least one febrile episode of ≥38 °C	6	67%
Days with fever, *n*, median (range)	1	(1–3)
Infections, *n* (%)		
Patients with at least one identified pathogen	4	44%
Bacteria, gram-positive	1	
Viral infection	3	

CRP: C-reactive protein; IL-6: Interleukin 6; CRS: Cytokine release syndrome. Bacteria: staphylococcus; Viral: Enterovirus (two patients), Varicella Zoster Virus (one patient).

**Table 4:** Outcomes of Glofitamab treatment.

Outcome Parameter			
Follow-up, median days (range)		246	(15–482)
Patients without relapse at last follow-up, no (%)		4/9 (44%)	
-PFS, days, median		161	
Relapse, *n* (%)		2	22%
-Time since glofitamab, days, median (range)		142	(123–161)
Patients alive at last follow-up, no (%)		5/9 (56%)	
Median OS		n.r.	
Deaths, *n* (% of all pts)		4	44%
-Time since glofitamab, days, median (range)		70	(15–246)
-Due to progression, *n* (%)		4	44%
Best overall response rate (ORR), %		67%	
Best CR rate, %		44%	
**Remission status**	First response	Best response	Last follow-up
CR	1	4	4
PR	5	2	0
SD	1	1	0
PD	2	2	5

OS: Overall survival; PFS: Progression-free survival; CR: Complete response; PR: Partial response; SD: Stable disease; PD: Progressive disease; First response: After cycle 1, 2 or 3. n.r.: not reached.

## Data Availability

Data can be acquired from the corresponding author by email.
